# Vision-Based SLAM System for Unmanned Aerial Vehicles

**DOI:** 10.3390/s16030372

**Published:** 2016-03-15

**Authors:** Rodrigo Munguía, Sarquis Urzua, Yolanda Bolea, Antoni Grau

**Affiliations:** 1 Department of Automatic Control, Technical University of Catalonia UPC, Barcelona 08036, Spain; yolanda.bolea@upc.edu; 2 Department of Computer Science, CUCEI, University of Guadalajara, Guadalajara 44430, Mexico; isi.sarquis@gmail.com

**Keywords:** state estimation, unmanned aerial vehicle, monocular vision, localization, mapping

## Abstract

The present paper describes a vision-based simultaneous localization and mapping system to be applied to Unmanned Aerial Vehicles (UAVs). The main contribution of this work is to propose a novel estimator relying on an Extended Kalman Filter. The estimator is designed in order to fuse the measurements obtained from: (i) an orientation sensor (AHRS); (ii) a position sensor (GPS); and (iii) a monocular camera. The estimated state consists of the full state of the vehicle: position and orientation and their first derivatives, as well as the location of the landmarks observed by the camera. The position sensor will be used only during the initialization period in order to recover the metric scale of the world. Afterwards, the estimated map of landmarks will be used to perform a fully vision-based navigation when the position sensor is not available. Experimental results obtained with simulations and real data show the benefits of the inclusion of camera measurements into the system. In this sense the estimation of the trajectory of the vehicle is considerably improved, compared with the estimates obtained using only the measurements from the position sensor, which are commonly low-rated and highly noisy.

## 1. Introduction

In recent years, many researchers have addressed the issue of making Unmanned Aerial Vehicles (UAVs) more autonomous. In this context, the state estimation of the six degrees of freedom (6-DoF) of a vehicle (*i.e*., its attitude and position) is a fundamental necessity for any application involving autonomy.

Outdoors, this problem is seemingly solved with on-board Global Positioning System (GPS) and Inertial Measurements Units (IMU) with their integrated version, the Inertial Navigation Systems (INS). In fact, unknown, cluttered, and GPS-denied environments still pose a considerable challenge. While attitude estimation is well-handled with available systems [1], GPS-based position estimation has some drawbacks. Specifically GPS is not a reliable service as its availability can be limited in urban canyons and is completely unavailable in indoor environments.

Moreover, even when GPS signal is available, the problem of position estimation could not be solved in different scenarios. For instance, aerial inspection of industrial plants is an application that requires performing precision manoeuvres in a complex environment. In this case, and due to the several sources of error, the position obtained with a GPS can vary with an error of several meters in just a few seconds for a static location [2]. In such a scenario, the use of GPS readings, smoothed or not, as the feedback signal of a control system can be unreliable because the control system cannot distinguish between sensor noise and actual small movements of the vehicle. Therefore, some additional sensory information (e.g., visual information) should be integrated into the system in order to improve accuracy.

The aforementioned issues have motivated the move of recent works towards the use of cameras to perform visual-based navigation in periods or circumstances when the position sensor is not available, when it is partially available, or when a local navigation application requires high precision. Cameras are well adapted for embedded systems because they are cheap, lightweight, and power-saving. In this way, a combination of vision and inertial measurements is often chosen as means to estimate the vehicle attitude and position. This combination can be performed with different approaches, as in [3], where the vision measurement is provided by an external trajectometry system, directly yielding the position and orientation of the robot. In [4], an external CCD camera provides the measurements. Other on-board techniques were proposed by [5,6], where an embedded camera uses different markers to provide a good estimation of position and orientation as well. This estimation was obtained using the specific geometry of different markers and assuming that the marker’s position was known. The same idea was exploited by [7], implemented with the low-cost Wii remote visual sensor. Finally, visual sensing is sometimes provided by optical flow sensors to estimate the attitude, the position, and the velocity, as in [8]. In these different approaches, position estimation is obtained by computer vision and the attitude is either obtained by vision (see [3,6]) or by IMU sensors. In [9], even a single angular measurement could significantly improve attitude and position estimation.

Another family of approaches (for instance [10,11]) relies on visual SLAM (Simultaneous Localization and Mapping) methods. In this case, the mobile robot operates in a *priori* unknown environment using only on-board sensors to simultaneously build a map of its surroundings and locate itself inside this map.

Robot sensors have a large impact on the algorithm used in SLAM. Early SLAM approaches focused on the use of range sensors, such as sonar rings and lasers, see [12,13,14,15]. Nevertheless, some disadvantages appear when using range sensors in SLAM: correspondence or data association becomes difficult, the sensors are expensive and have a limited working range, and some of them are limited to 2D maps. For small unmanned aerial vehicles, there exist several limitations regarding the design of the platform, mobility, and payload capacity that impose considerable restrictions. Once again, cameras appear as a good option to be used in SLAM systems applied to UAVs.

In this work, the authors propose the use of a monocular camera looking downwards, integrated into the aerial vehicle, in order to provide visual information of the ground. With such information, the proposed visual-based SLAM system will be using visual information, attitude, and position measurements in order to accurately estimate the full state of the vehicle as well as the position of the landmarks observed by the camera.

Compared with another kind of visual configurations (e.g., stereo vision), the use of monocular vision has some advantages in terms of weight, space, power consumption, and scalability. For example, in stereo rigs, the fixed base-line between cameras can limit the operation range. On the other hand, the use of monocular vision introduces some technical challenges. First, depth information cannot be retrieved in a single frame, and hence, robust techniques to recover features depth are required. In this work, a novel method is developed following the research initiated in [16]. The proposed approach is based on a stochastic technique of triangulation to estimate features depth.

In this novel research, a new difficulty appears: the metric scale of the world cannot be retrieved if monocular vision is used as the unique sensory input to the system. For example, in the experiments presented in [17], the first ten measurements are aligned with the ground-truth in order to obtain the scale of the environment. In [18], the monocular scale factor is retrieved from a feature pattern with known dimensions. On the other hand, in many real scenarios GPS signal is available, at least for some periods. For this reason, in this work it is assumed that the GPS signal is known during a short period (for some seconds) at the beginning of the trajectory. Those GPS readings will be integrated into the system in order to recover the metric scale of the world. This period of time is what authors consider the initialization period. After this period, the system can rely only on visual information to estimate the position of the aerial vehicle.

The integration of GPS readings with visual information is not new in the literature, see [19]. In this sense, one of the contributions of this work is to demonstrate that the integration of very noisy GPS measurements into the system for an initial short period is enough to recover the metric scale of the world. Furthermore, the experiments demonstrate that for flight trajectories performed near the origin of the navigation reference frame, it is better to avoid the integration of such GPS measurements after the initialization period.

This paper is organized as follows: Section 2 states the problem description and assumptions. Section 3 describes the proposed method in detail. Section 4 shows the experimental results, and finally in Section 5, the conclusions of this work are presented.

## 2. System Specification

### 2.1. Assumptions

**Figure 1:**
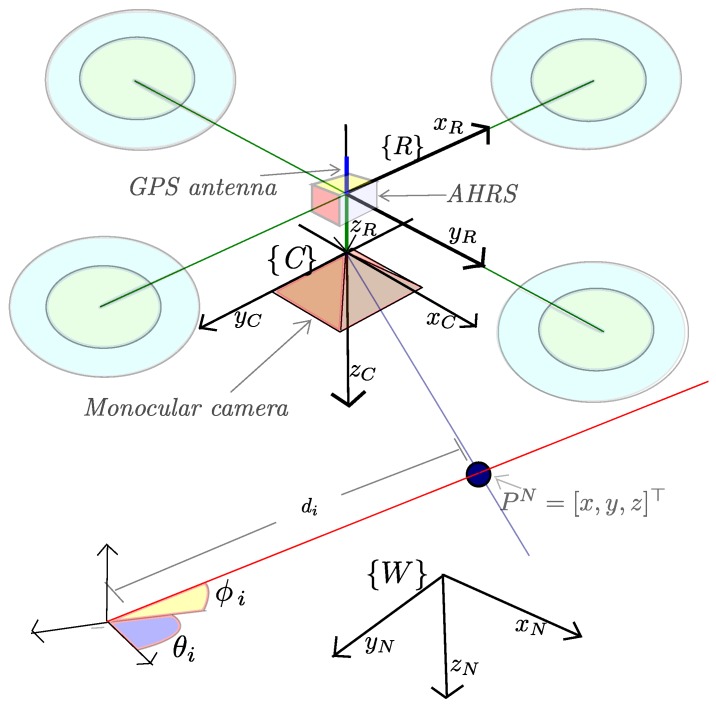
Coordinate systems: the local tangent frame is used as the navigation reference frame ${}^{N}$. AHRS: Attitude and Heading Reference System.

The platform that the authors consider in this work is a quadrotor freely moving in any direction in $R^{3}\times SO\left(3\right)$, as shown in Figure 1. The quadrotor is equipped with a monocular camera, an attitude and heading reference system (AHRS) and a position sensor (GPS). It is important to remark that the proposed visual-based SLAM approach can be applied to another kind of platforms.

The proposed system is mainly intended for local autonomous vehicle navigation. Hence, the local tangent frame is used as the navigation reference frame. The initial position of the vehicle defines the origin of the navigation coordinates frame. The navigation system follows the NED (North, East, Down) convention. In this work, the magnitudes expressed in the navigation, vehicle (robot), and camera frame are denoted respectively by the superscripts ${}^{N}$, ${}^{R}$, and ${}^{C}$. All the coordinate systems are right-handed defined.

In this research, the sensors that have been taken into account are described and modelled in the following subsections.

### 2.2. Monocular Camera

As a vision system, a standard monocular camera has been considered. In this case, a central-projection camera model is assumed. The image plane is located in front of the camera’s origin where a non-inverted image is formed. The camera frame ${}^{C}$ is right-handed with the *z*-axis pointing to the field of view.

The $R^{3}\Rightarrow R^{2}$ projection of a 3D point located at $p^{N}={\left(x,y,z\right)}^{T}$ to the image plane (u,v) is defined by:

(1) $$u=\frac{x^{\prime }}{z^{\prime }}\;\;\;\;\;v=\frac{y^{\prime }}{z^{\prime }}$$

where *u* and *v* are the coordinates of the image point *p* expressed in pixel units, and:

(2) $$\left[\begin{array}{c} x^{\prime }\\ y^{\prime }\\ z^{\prime } \end{array}\right]=\left[\begin{array}{ccc} f & 0 & u_{0}\\ 0 & f & v_{0}\\ 0 & 0 & 1 \end{array}\right]p^{C}$$

being $p^{C}$ the same 3D point $p^{N}$, but expressed in the camera frame ${}^{C}$ by $p^{C}=R^{NC}\left(p^{N}-t_{c}^{N}\right)$. In this case, it is assumed that the intrinsic parameters of the camera are already known: (i) focal length *f*; (ii) principal point $u_{0},v_{0}$; and (iii) radial lens distortion $k_{1},...,k_{n}$.

Let $R^{NC}={\left(R^{RN}R^{CR}\right)}^{T}$ be the rotation matrix that transforms the navigation frame ${}^{N}$ to the camera frame ${}^{C}$. Let $R^{CR}$ be a known value, and $R^{RN}$ is computed from the current robot quaternion $q^{NR}$. Let $t_{c}^{N}=r^{N}+R^{RN}t_{c}^{R}$ be the position of the camera’s optical center position expressed in the navigation frame.

Inversely, a directional vector $h^{C}={\left[h_{x}^{C},h_{y}^{C},h_{z}^{C}\right]}^{T}$ can be computed from the image point coordinates *u* and *v*.

(3) $$h^{C}\left(u,v\right)={\left[\frac{u_{0}-u}{f},\frac{v_{0}-v}{f},1\right]}^{T}$$

The vector $h^{C}$ points from the camera optical center position to the 3D point location. $h^{C}$ can be expressed in the navigation frame by $h^{N}=R^{CN}h^{C}$, where $R^{CN}=R^{RN}R^{CR}$ is the camera-to-navigation rotation matrix. Note that for the $R^{2}\Rightarrow R^{3}$ mapping case, defined in Equation (3), depth information is lost.

The distortion caused by the camera lens is considered through the model described in [20]. Using this model (and its inverse form), undistorted pixel coordinates (u,v) can be obtained from the distorted pixel $\left(u_{d},v_{d}\right)$, and conversely.

### 2.3. Attitude and Heading Reference System

An attitude and heading reference system (AHRS) is a combination of instruments capable of maintaining an accurate estimation of the vehicle attitude while it is manoeuvring. Recent manufacturing of solid-state or MEMS gyroscopes, accelerometers, magnetometers, and powerful microcontrollers as well, have made possible the development of small, low-cost, and reliable AHRS devices (e.g., [1,21,22]). For these reasons, in this work a loosely-coupled approach is considered. In this case, the information of orientation provided by the AHRS is explicitly fused into the system. Hence, the availability of high-rated (typically 50–100 Hz) attitude measurements provided by a decoupled AHRS device are assumed.

Attitude measurements $y_{a}^{N}$ are modelled by:

(4) $$y_{a}^{N}=a^{N}+v_{a}$$

where $a^{N}={\left[\phi_{v},\theta_{v},\psi_{v}\right]}^{T}$, being $\phi_{v}$, $\theta_{v}$, and $\psi_{v}$ Euler angles denoting respectively the roll, pitch, and yaw of the vehicle. Let $v_{a}$ be a Gaussian white noise with power spectral density (PSD) $\sigma_{a}^{2}$.

### 2.4. GPS

**Figure 2:**
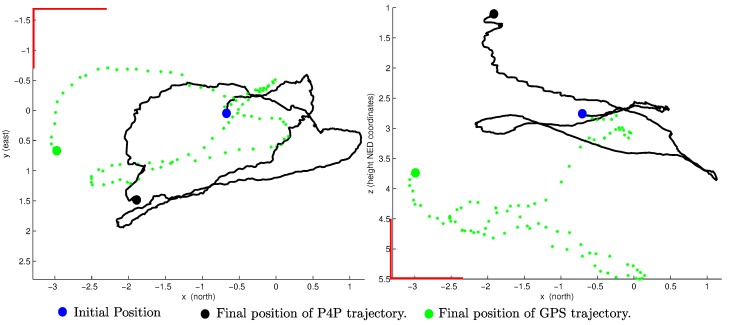
Example of GPS position measurements obtained for a flight performed by the aerial vehicle. Top view (**left plot**) and lateral view (**right plot**) are shown for clarity. Flight trajectory has been computed using the perspective on 4-point (P4P) method described in Section 4. Error drift in GPS readings is noticeable. NED: North, East, Down.

The Global Positioning System (GPS) is a satellite-based navigation system that provides 3D position information for objects on or near the Earth’s surface. The GPS system and global navigation satellite systems have been described in detail in numerous studies (e.g., [2,23]). Several sources of error affect the accuracy of GPS position measurements. The cumulative effect of each of these error sources is called the user-equivalent range error (UERE). In [2], these errors are characterized as a combination of slowly varying biases and random noise. In [24] it is stated that the total UERE is approximately 4.0 m (*σ*), from which 0.4 m (*σ*) correspond to random noise. In Figure 2, a comparison between the trajectory obtained with GPS and the actual one, flying in a small area, is shown.

In this work, it is assumed that position measurements $y_{r}$ can be obtained from the GPS unit, at least at the beginning of the trajectory, and they are modelled by:

(5) $$y_{r}=r^{N}+v_{r}$$

where $v_{r}$ is a Gaussian white noise with PSD $\sigma_{r}^{2}$, and $r^{N}$ is the position of the vehicle.

Commonly, position measurements are obtained from GPS devices in geodetic coordinates (*latitude*, *longitude*, and *height*). Therefore, in Equation (5) it is assumed that GPS position measurements have been previously transformed to their corresponding local tangent frame coordinates. It is also assumed that the offset between the GPS antenna and the vehicle frame has been taken into account in the previous transformation.

### 2.5. Sensor Fusion Approach

The estimator proposed in this work is designed in order to estimate the full state of the vehicle, which will contain the position and orientation of the vehicle and their first derivatives, as well as the location of the landmarks observed by the camera.

Attitude estimation can be well-handled by the available systems in the vehicle, as has been mentioned in the above subsections. Typically, the output of the AHRS is directly used as a feedback to the control system for stabilizing the flying vehicle. On the other hand, the proposed method requires the camera–vehicle to know its orientation in order to estimate its position, as will be discussed later in the paper. In order to account for the uncertainties associated with the estimation provided by the AHRS, the orientation is included into the state vector (see Section 3.1) and is explicitly fused into the system (see Section 3.4).

Regarding the problem of position estimation, it cannot be solved for applications that require performing precise manoeuvres, even when GPS signal is available, as it can be inferred from the example presented in Section 2.4. Therefore, some additional sensory information (e.g., monocular vision) should be integrated into the system in order to improve its accuracy. On the other hand, one of the most challenging aspects of working with monocular sensors has to do with the impossibility of directly recovering the metric scale of the world. If no additional information is used, and a single camera is used as the sole source of data to the system, the map and trajectory can only be recovered without metric information [25].

Monocular vision and GPS are not suitable to be used separately for navigation purposes in some scenarios. For this reason, the noisy data obtained from the GPS is added during the initialization period in order to incorporate metric information into the system. Hence, after an initial period of convergence, where the system is considered to be in the initialization mode, the system can operate relying only on visual information to estimate the vehicle position.

## 3. Method Description

### 3.1. Problem Description

The main goal of the proposed method is to estimate the following system state *x*:

(6) $$x={\left[x_{v},y_{1}^{N},y_{2}^{N},...,y_{n}^{N}\right]}^{T}$$

where $x_{v}$ represents the state of the unmanned aerial vehicle, and $y_{i}^{N}$ represents the location of the *i*-th feature point in the environment. At the same time, $x_{v}$ is composed of:

(7) $$x_{v}={\left[q^{NR},\omega^{R},r^{N},v^{N}\right]}^{T}$$

where $q^{NR}=\left[q_{1},q_{2},q_{3},q_{4}\right]$ represents the orientation of the vehicle respect to the world (navigation) frame by a unit quaternion. Let $\omega^{R}=\left[\omega_{x},\omega_{y},\omega_{z}\right]$ be the angular velocity of the robot expressed in the same frame of reference. Let $r^{N}=\left[p_{x},p_{y},p_{z}\right]$ represent the position of the vehicle (robot) expressed in the navigation frame. Let $v^{N}=\left[v_{x},v_{y},v_{z}\right]$ denote the linear velocity of the robot expressed in the navigation frame. The location of a feature $y_{i}^{N}$ is parametrized in its euclidean form:

(8) $$y_{i}^{N}={\left[p_{x_{i}},p_{y_{i}},p_{z_{i}}\right]}^{T}$$

In the remainder of the paper, the superscript ${}^{N}$ will be dropped from $y_{i}^{N}$ to avoid confusion.

**Figure 3:**
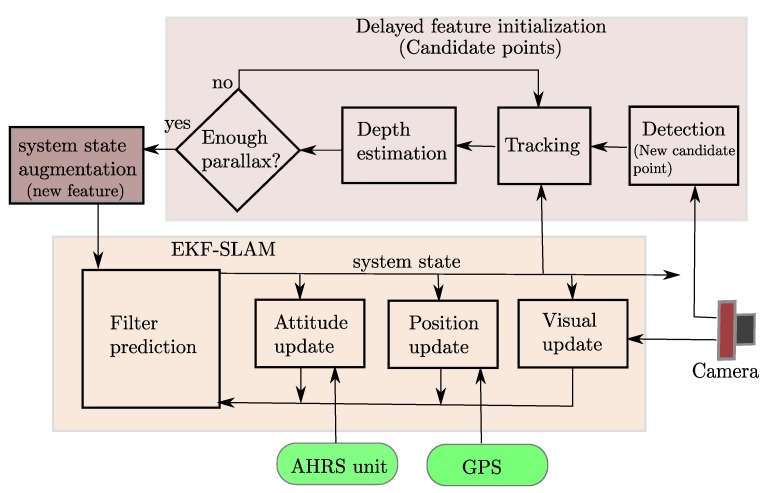
Block diagram showing the architecture of the system. EKF-SLAM: Extended Kalman Filter Simultaneous Localization and Mapping.

The architecture of the system is defined by the a classical loop of prediction-update steps in the Extended Kalman Filter (EKF) in its direct configuration. In this case, the vehicle state as well as the feature estimates are propagated by the filter, see Figure 3.

### 3.2. Prediction

At the same frequency of the AHRS operation, the vehicle system state $x_{v}$ takes a step forward through the following unconstrained constant-acceleration (discrete) model:

(9) $$\left\{\begin{array}{c} q_{k+1}^{NC}=\left(\cos∥w∥I_{4\times 4}+\frac{\sin∥w∥}{∥w∥}W\right)q_{k}^{NC}\\ \omega_{k+1}^{C}=\omega_{k}^{C}+\Omega^{C}\\ r_{k+1}^{N}=r_{k}^{N}+v_{k}^{N}\Delta t\\ v_{k+1}^{N}=v_{k}^{N}+V^{N} \end{array}\right.$$

In the model represented by Equation (9), a closed form solution of $\dot{q}=1/2\left(W\right)q$ is used to integrate the current velocity rotation $\omega^{C}$ over the quaternion $q^{NC}$. In this case $w={\left[\omega_{k+1}^{C}\Delta t/2\right]}^{T}$ and:

(10) $$W=\left[\begin{array}{cccc} 0 & -w_{1} & -w_{2} & -w_{3}\\ w_{1} & 0 & -w_{3} & w_{2}\\ w_{2} & w_{3} & 0 & -w_{1}\\ w_{3} & -w_{2} & w_{1} & 0 \end{array}\right]$$

At every step, it is assumed that there is an unknown linear and angular velocity with acceleration zero-mean and known-covariance Gaussian processes $\sigma_{v}$ and $\sigma_{\omega }$, producing an impulse of linear and angular velocity: $V^{N}=\sigma_{v}^{2}\Delta t$ and $\Omega^{C}=\sigma_{\omega }^{2}\Delta t$.

It is assumed that the map features $y_{i}$ remain static (rigid scene assumption) so $x_{k+1}={\left[x_{v(k+1)},y_{1(k)},y_{2(k)},...,y_{n(k)}\right]}^{T}$.

The state covariance matrix ***P*** takes a step forward by:

(11) $$P_{k+1}=\nabla F_{x}P_{k}\nabla F_{x}^{T}+\nabla F_{u}Q\nabla F_{u}^{T}$$

where Q and the Jacobians $\nabla F_{x}$, $\nabla F_{u}$ are defined as:

(12) $$\nabla F_{x}=\left[\begin{array}{cc} \frac{\partial f_{v}}{\partial {\hat{x}}_{v}} & 0_{13\times n}\\ 0_{n\times 13} & I_{n\times n} \end{array}\right],\nabla F_{u}=\left[\begin{array}{cc} \frac{\partial f_{v}}{\partial u} & 0_{13\times n}\\ 0_{n\times 6} & 0_{n\times n} \end{array}\right],Q=\left[\begin{array}{cc} U & 0_{6\times n}\\ 0_{n\times 6} & 0_{n\times n} \end{array}\right]$$

Let $\frac{\partial f_{v}}{\partial x_{v}}$ be the derivatives of the equations of the nonlinear prediction model (Equation (9)) with respect to the robot state $x_{v}$. Let $\frac{\partial f_{v}}{\partial u}$ be the derivatives of the nonlinear prediction model with respect to the unknown linear and angular velocity. The Jacobian calculation is a complicated but tractable matter of differentiation, hence, no results are presented here. Uncertainties are incorporated into the system by means of the process noise covariance matrix $U=diag[\sigma_{a}^{2}I_{3\times 3},\sigma_{\omega }^{2}I_{3\times 3}]$, through parameters $\sigma_{a}^{2}$ and $\sigma_{\omega }^{2}$.

### 3.3. Visual Aid

Depth information cannot be obtained in a single measurement when bearing sensors (e.g., a projective camera) are used. To infer the depth of a feature, the sensor must observe this feature repeatedly as the sensor freely moves through its environment, estimating the angle from the feature to the sensor center. The difference between those angle measurements is the parallax angle. Actually, parallax is the key that allows the estimation of features depth. In the case of indoor sequences, a displacement of centimeters could be enough to produce parallax; on the other hand, the more distant the feature, the more the sensor has to travel to produce parallax.

In monocular-based systems, the treatment of the features in the stochastic map (initialization, measurement, *etc*.) is an important problem to address with direct implications in the robustness of the system. In this work, a novel method is proposed in order to incorporate new features into the system. In this approach, a single hypothesis is computed for the initial depth of features by means of a stochastic technique of triangulation. The method is based on previous authors’ work [16], and new contributions have been introduced in this research.

#### 3.3.1. Detection of Candidate Points

**Figure 4:**
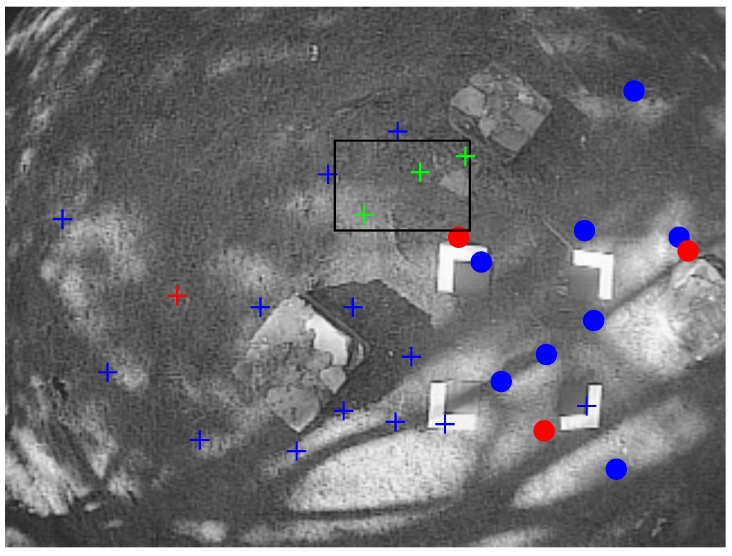
Candidate points are detected randomly in image regions without map features or candidate points. In this frame, the black rectangle indicates the current search region. Three new candidate points have been detected (green cross-marks). Candidate points being tracked are indicated by blue cross-marks. Visual features already mapped are indicated by circles. Red marks indicate unsuccessful matches.

The proposed method states that a minimum number of features $y_{i}$ is considered to be predicted appearing in the image, otherwise new features should be added to the map. In this latter case, new points are detected in the image through a random search. For this purpose, Shi-Tomasi corner detector [26] is applied, but other detectors could be also used. These points in the image, which are not yet added to the map, are called candidate points, see Figure 4. Only image areas free of both candidate points and mapped features are considered to detect new points with the saliency operator.

At the *k* frame, when a visual feature is detected for the first time, the following entry $c_{l}$ is stored in a table:

(13) $$c_{l}=\left[{\left(t_{c_{0}}^{N}\right)}^{T},\theta_{0},\phi_{0},P_{y_{c_{i}}},z_{uv}\right]$$

where $z_{uv}=\left[u,v\right]$ is the location in the image of the candidate point. Let $y_{c_{i}}={\left[t_{c_{0}}^{N},\theta_{0},\phi_{0}\right]}^{T}=h\left(\hat{x},z_{uv}\right)$ be a variable that models a 3D semi-line, defined on one side by the vertex $t_{c_{0}}^{N}$, corresponding to the current optical center coordinates of the camera expressed in the navigation frame, and pointing to infinity on the other side, with azimuth and elevation $\theta_{0}$ and $\phi_{0}$, respectively, and:

(14) $$\begin{array}{c} \theta_{0}=\mathrm{atan}2\left(h_{y}^{N},h_{x}^{N}\right)\\ \phi_{0}=\mathrm{acos}\left(\frac{h_{z}^{N}}{\sqrt{{\left(h_{x}^{N}\right)}^{2}+{\left(h_{y}^{N}\right)}^{2}+{\left(h_{z}^{N}\right)}^{2}}}\right) \end{array}$$

where $h^{N}={\left[h_{x}^{N},h_{y}^{N},h_{z}^{N}\right]}^{T}$ is computed as indicated in Section 2.2. $P_{y_{i}}$ is a 5×5 covariance matrix which models the uncertainty of $y_{c_{i}}$ in the form of $P_{y_{c_{i}}}=\nabla Y_{c_{i}}P\nabla Y_{c_{i}}^{T}$, where *P* is the system covariance matrix and $\nabla Y_{c_{i}}$ is the Jacobian matrix formed by the partial derivatives of the function $y_{c_{i}}=h\left(\hat{x},z_{uv}\right)$ with respect to ${\left[\hat{x},z_{uv}\right]}^{T}$.

Also, a p×p pixel window, centered in [u,v] is extracted and related to the corresponding candidate point.

#### 3.3.2. Tracking of Candidate Points

To infer the depth of a feature, the sensor must observe this feature repeatedly until a minimum parallax is reached. For this reason, it is necessary to have a method to track the location in the image of candidate points whose initial depth must be computed. For feature points that have already been included into the system state, there is enough information (e.g., depth) to define probability regions where these points must lie based on the statistical information available in the system state (see [27]). On the other hand, for candidate points, there is not yet information about depth nor statistical correlations with other elements of the system. In this sense, one alternative is to use a general-purpose decoupled tracking method that works on the images and does not need assumptions about the system dynamics (e.g., [26]). Due to the lack of information about system dynamics, these kinds of methods usually define regions of search with symmetric geometry and fixed size. This factor can add some extra computational cost.

In this work, a novel technique to track candidate points is proposed. The idea is to take advantage of the knowledge about the direction of the movement of the camera in order to define regions of search defined by very thin ellipses. The ellipses are aligned with the epipolar lines where the candidate points must lie.

**Figure 5:**
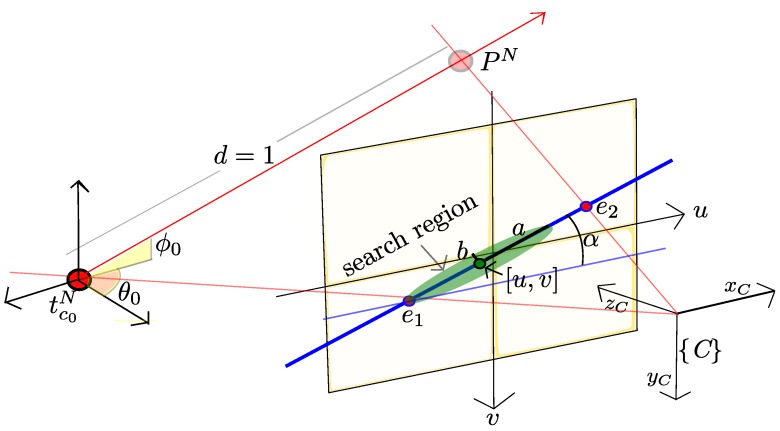
The established search region to match candidate points is constrained to ellipses aligned with the epipolar line.

At every subsequent frame k+1,k+2...k+n, the location of candidate points is tracked. In this case, a candidate point is predicted to be appearing inside an elliptical region $S_{c}$ centered in the point [u,v], taken from $c_{l}$, see Figure 5. In order to optimize the speed of the search, the major axis of the ellipse is aligned with the epipolar line defined by image points $e_{1}$ and $e_{2}$.

The epipole $e_{1}$ is computed by projecting $t_{c_{0}}^{N}$, which is stored in $c_{l}$, to the current image plane by Equations (1) and (2). Because there is not depth information of the candidate point, an hypothetical depth equal to one (d=1) is chosen in order to determine a virtual 3D point $p^{N}$ which lies in the semi-ray defined by $c_{l}$. The epipole $e_{2}$ is then computed by projecting this virtual 3D point $p^{N}$ through Equations (1) and (2).

In this case, $p^{N}$ will model a 3D point located at:

(15) $$p^{N}=t_{c_{0}}^{N}+m\left(\theta_{0},\phi_{0}\right)d$$

where $m(\theta_{0},\phi_{0})$ is a directional unitary vector defined by: $m\left(\theta_{0},\phi_{0}\right)={\left(\cos\theta_{0}\sin\phi_{0},\sin\theta_{0}\sin\phi_{0},\cos\phi_{0}\right)}^{T}$.

The orientation of the ellipse $S_{c}$ is determined by $\alpha_{c}=\mathrm{atan}2\left(e_{y},e_{x}\right)$, where $e_{y},e_{x}$ represents the *y* and *x* coordinates, respectively, of *e*, and $e=e_{2}-e_{1}$. The size of the ellipse $S_{c}$ is determined by its major and minor axis, respectively *a* and *b*.

The ellipse $S_{c}$ is represented in its matrix form by:

(16) $$\begin{array}{c} S_{c}=R_{c}\left[\begin{array}{cc} a & 0\\ 0 & b \end{array}\right]R_{c}^{T}\\ R_{c}=\left[\begin{array}{cc} \cos\alpha_{c} & -\sin\alpha_{c}\\ \sin\alpha_{c} & \cos\alpha_{c} \end{array}\right] \end{array}$$

The ellipse $S_{c}$ represents a probability region where the candidate point must lie in the current frame. The proposed tracking method is intended to be used during an initial short period of time. During this period, some information will be gathered in order to compute a depth hypothesis for each candidate point, prior to its initialization as a new map feature. For this reason, there is no extra effort to obtain more robust variations in scale or a rotations descriptor. In this case, direct patch cross-correlation is applied over all the image locations $\left[u_{i},v_{i}\right]\in S_{c}$ . If the score of a location $\left[u_{i},v_{i}\right]$, determined by the best cross-correlation between the candidate patch and the *n* patches defined by the region of search, is higher than a specific threshold, then this pixel location $\left[u_{i},v_{i}\right]$ is considered as the current candidate point location. Thus, $c_{l}$ is updated with $z_{uv}=\left[u_{i},v_{i}\right]$.

Unfortunately, because there is not yet reliable information about the depth of candidate points, it is difficult to determine an optimal and adaptive size of the ellipse. In this case, *a* is left as a free parameter to be chosen empirically as a function of the particularities of the application (e.g., maximum velocity of the vehicle, video frame rate). For the application presented in this work, good results were found with a value of a=20 pixels.

**Figure 6:**
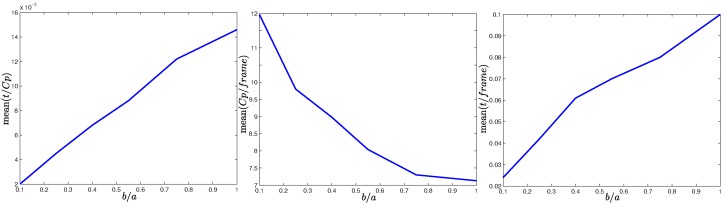
Results obtained by means of the variation of the relation between ellipse $S_{c}$ axes (b/a). (**left plot**): average tracking time for a candidate point; (**middle plot**): average number of candidate points being tracked at each frame; (**right plot**): average total time per frame. These results were obtained using the same methodology described in Section 4.2.

On the other hand, it is possible to investigate the effects obtained by the variation of the relation of (b/a) which determines the proportion of the ellipse. In Figure 6, it can be noted that the time required to track a candidate point increases considerably as the ellipse tends to be a circle (left plot). On the other hand, the number of candidate points being tracked is lower when the ellipse tends to be a circle (middle plot). This is because some candidate points are lost when the ellipse is too thin, and new candidate points must be detected. Even so, the total time required for the whole tracking process of candidate points is much lower when the parameter *b* is chosen in order to define a very thin ellipse (right plot). For the foregoing reason the value of parameter *b* is recommended to be ten times lower than *a*.

#### 3.3.3. Estimating Candidate Points Depth

Every time that a new image location $z_{uv}=\left[u,v\right]$ is obtained for a candidate point $c_{l}$, an hypothesis of depth $d_{i}$ is computed by:

(17) $$d_{i}=\frac{∥e_{l}∥\sin\gamma }{\sin\alpha_{i}}$$

Let $\alpha_{i}=\pi -\left(\beta +\gamma \right)$ be the parallax. Let $e_{l}=t_{c_{0}}^{N}-t_{c}^{N}$ indicate the displacement of the camera from the first observation position to its current position, with:

(18) $$\beta =\cos^{-1}\left(\frac{h_{1}\;\cdot \;e_{l}}{∥h_{1}∥∥e_{l}∥}\right)\;\;\;\;\;\gamma =\cos^{-1}\left(\frac{-h_{2}\;\cdot \;e_{l}}{∥h_{2}∥∥e_{l}∥}\right)$$

**Figure 7:**
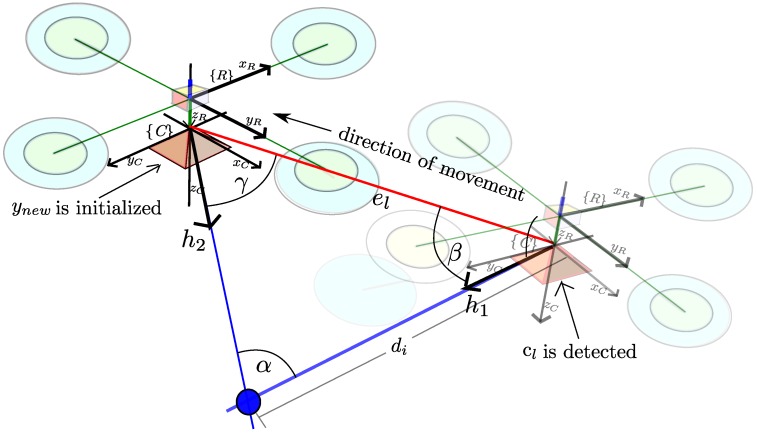
An hypothesis $d_{i}$ for the depth of a candidate point is computed by triangulating between the first location when the point was detected and the current location of the vehicle.

Let *β* be the angle defined by $h_{1}$ and $e_{l}$. Let $h_{1}$ be the normalized directional vector $m\left(\theta_{i},\phi_{i}\right)={\left(\cos\theta_{i}\sin\phi_{i},\sin\theta_{i}\sin\phi_{i},\cos\phi_{i}\right)}^{T}$ computed taking $\theta_{i}$, $\phi_{i}$ from $c_{l}$, and where *γ* is the angle defined by $h_{2}$ and $-e_{l}$. Let $h_{2}=h^{N}$ be the directional vector pointing from the current camera optical center to the feature location, computed as indicated in Section 2.2 from the current measurement $z_{uv}$, see Figure 7.

At each step, there may be a considerable variation in depth computed by triangulation, specially for low parallax. In previous authors’ work [28], it is shown that estimates are greatly improved by filtering the hypotheses of depth $d_{i}$ with a simple low-pass filter. Moreover, in this work it is demonstrated that only a few degrees of parallax is enough to reduce the uncertainty in the depth estimation. When parallax $\alpha_{i}$ is greater than a specific threshold ($\alpha_{i}>\alpha_{min}$) a new feature $y_{new}={\left[p_{x_{i}},p_{y_{i}},p_{z_{i}}\right]}^{T}=h\left(c_{l},d\right)$ is added to the system state vector *x*:

(19) $$x_{new}={\left[x_{old};y_{new}\right]}^{T}$$

where

(20) $$y_{new}=t_{c_{0}}^{N}+m\left(\theta_{i},\phi_{i}\right)d_{i}$$

The system state covariance matrix *P* is updated by:

(21) $$P_{new}=\left[\begin{array}{cc} P_{old} & 0\\ 0 & P_{y_{new}} \end{array}\right]$$

where $P_{y_{new}}$ is the 3×3 covariance matrix which models the uncertainty of the new feature $y_{new}$, and:

(22) $$P_{y_{new}}=\nabla Y\left[\begin{array}{cc} P_{y_{i}} & 0\\ 0 & \sigma_{d}^{2} \end{array}\right]\nabla Y^{T}$$

In Equation (22), $P_{y_{i}}$ is taken from $c_{l}$ (Equation (13)). Let $\sigma_{d}^{2}$ be a parameter modelling the uncertainty of process of depth estimation. Let ∇Y be the Jacobian matrix formed by the partial derivatives of the function $y_{new}=h\left(c_{l},d\right)$ with respect to ${\left[{\left(t_{c_{0}}^{N}\right)}^{T},\theta_{0},\phi_{0},d\right]}^{T}$.

#### 3.3.4. Visual Updates and Map Management

The process of tracking visual features $y_{i}$ is conducted by means of an active search technique [27]. In this case, and in a different way from the tracking method described in Section 3.3.2, the search region is defined by the innovation covariance matrix $S_{i}$, where $S_{i}=\nabla H_{i}P_{k+1}\nabla H_{i}^{T}+\xi_{i}$.

Assuming that for the current frame, *n* visual measurements are available for features $y_{1},y_{2},...,y_{n}$, then the filter is updated with the Kalman update equations as follows:

(23) $$\left\{\begin{array}{c} x_{k}=x_{k+1}+K\left(z-h\right)\\ P_{k}=P_{k+1}-KSK^{T}\\ K=P_{k+1}\nabla H^{T}S^{-1}\\ S=\nabla HP_{k+1}\nabla H^{T}+\xi \end{array}\right.$$

where $z={\left[z_{{uv}_{1}},z_{{uv}_{2}},...,z_{{uv}_{n}}\right]}^{T}$ is the current measurement vector. Let $h={\left[h_{1},h_{2},...,h_{n}\right]}^{T}$ be the current prediction measurement vector. The measurement prediction model $h_{i}=\left(u,v\right)=h\left(x_{v},y_{i}\right)$ has been defined in Section 2.2. Let *K* be the Kalman gain. Let *S* be the innovation covariance matrix. Let $\nabla H={\left[\nabla H_{1},\nabla H_{2},...\nabla H_{n}\right]}^{T}$ be the Jacobian formed by the partial derivatives of the measurement prediction model h(x) with respect to the state *x*.

(24) $$\nabla H_{i}=\left[\frac{\partial h_{i}}{\partial x_{v}},...0_{2\times 3}...\frac{\partial h_{i}}{\partial y_{i}},...0_{2\times 3}...\right]$$

Let $\frac{\partial h_{i}}{\partial x_{v}}$ be the partial derivatives of the equations of the measurement prediction model $h_{i}$ with respect to the robot state $x_{v}$. Let $\frac{\partial h_{i}}{\partial y_{i}}$ be the partial derivatives of $h_{i}$ with respect to feature $y_{i}$. Note that $\frac{\partial h_{i}}{\partial y_{i}}$ has only a nonzero value at the location (indexes) of the observed feature $y_{i}$. Let $\xi =\left(I_{2n\times 2n}\right)\sigma_{uv}^{2}$ be the measurement noise covariance matrix. Let $\sigma_{uv}^{2}$ be the variance modelling the uncertainty in visual measurements.

A SLAM framework that works reliably in a local way can easily be applied to large-scale problems using different methods, such as sub-mapping, graph-based global optimization [29], or global mapping [30]. Therefore, in this work, large-scale SLAM and loop-closing are not considered. However, these problems have been intensively studied in the past. Candidate points whose tracking process is failing are pruned from the system. Furthermore, visual features with high percentage of mismatching are removed from the system state and covariance matrix. The removal process is carried out using the approach described in [31].

### 3.4. Attitude and Position Updates

When an attitude measurement $y_{a}^{N}$ is available, the system state is updated. Since most low-cost AHRS devices provide their output in Euler angles format, the following measurement prediction model $h_{a}=h\left({\hat{x}}_{v}\right)$ is used:

(25) $$\left[\begin{array}{c} \theta_{v}\\ \phi_{v}\\ \psi_{v} \end{array}\right]=\left[\begin{array}{c} \mathrm{atan}2(2\left(q_{3}q_{4}-q_{1}q_{2}\right),1-2\left(q_{2}^{2}+q_{3}^{2}\right))\\ \mathrm{asin}(-2\left(q_{1}q_{3}+q_{2}q_{4}\right))\\ \mathrm{atan}2(2\left(q_{2}q_{3}-q_{1}q_{4}\right),1-2\left(q_{3}^{2}+q_{4}^{2}\right)) \end{array}\right]$$

During the initialization period, position measurements $y_{r}$ are incorporated into the system using the simple measurement model $h_{r}=h\left({\hat{x}}_{v}\right)$:

(26) $$h_{r}={\left[p_{x},p_{y},p_{z}\right]}^{T}$$

The regular Kalman update equations (Equation (23)) are used to update attitude and position whenever is required, but using the corresponding Jacobian ∇H and measurement noise covariance matrix *R*.

The metric scale of the world cannot be retrieved using only monocular vision, as mentioned previously, and thus additional information must be added to the system. For instance, the metric scale can be retrieved if the position of some landmarks are known *a priori* with low uncertainty [32]. In this work, it is assumed that the GPS signal is available for an initial period at least. This period is considered as an initialization period that must allow the convergence of depth for at least some features close to their actual values. These first features added to the map during the initialization period set a metric scale in estimations. Afterwards, the system can operate relying only on visual information to estimate the location of the vehicle.

For the proposed method, the initialization period will end when at least *n* features show a certain degree of convergence. It has been theoretically demonstrated (e.g., [33]) that knowledge about the position of three landmarks can be enough to make the metric scale observable. However, in practice, there is always the possibility that the tracking process of some features fails at any time. For this reason, in this work the initialization period will be ending when n≥3 features have converged. In experiments, good results have been found with n=5.

In [34], the convergence of features is tested using the Kullback distance. However, the complexity of the sampling method proposed to evaluate this distance is quite high. In the present work, good results have been found with the following criteria:

(27) $$\max\left(eig\left(P_{y_{i}}\right)\right)<\frac{∥y_{i}-r^{N}∥}{100}$$

where $P_{y_{i}}$ is the 3×3 sub-matrix extracted from the covariance matrix *P* corresponding to the $y_{i}$ feature. In this case, if the greater eigenvalue of $P_{y_{i}}$ is smaller than one percent of the distance between the camera and the feature, then it is considered that the uncertainty in this feature has been minimized enough to take it as an initial reference of metrics.

It is important to note that the origin of the local reference system of navigation is established at the end of the initialization period. The reason is because at the beginning of the movement the GPS errors can wrongly dominate the estimations.

**Figure 8:**
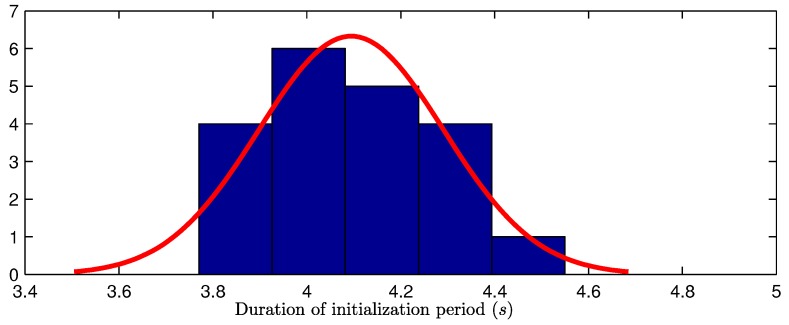
Histogram of the duration of the initialization period obtained after 20 runs of the proposed method. This particular case corresponds to the flight trajectory presented in Section 4.2.

Since the proposed method is not deterministic, the duration of the initialization period varies even for the same input dataset (see Figure 8). For this reason, in order to simplify the experimental methodology, a fixed initialization period was used for computing the results of comparative studies presented in Section 4. In this manner, it was easier to align (in time) the estimated trajectories in order to perform a Monte Carlo validation. The fixed initial period was empirically determined to allow a high percentage of initial convergence. In a real scenario, the duration of the initialization period should be determined by an adaptive criteria, as authors have proposed in this section.

## 4. Experimental Results

In this section, the results obtained using synthetic data from simulations are presented as well as the results obtained from experiments with real data. The experiments were performed in order to validate the performance of the proposed method. A MATLAB^®^ implementation was used for this purpose.

### 4.1. Experiments with Simulations

In simulations, the model used to implement the vehicle dynamics was taken from [35]. To model the transient behaviour of the GPS error, the approach of [36] was followed. The monocular camera was simulated using the same parameter values of the camera used in the experiments with real data. The parameter values used to emulate the AHRS were taken from [1].

**Figure 9:**
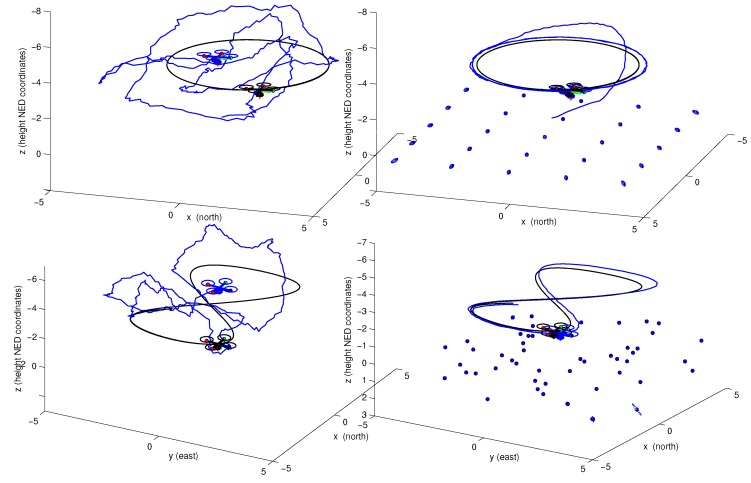
Comparison of the estimated trajectories obtained by filtering GPS data (**left plots**), and the estimated maps and trajectories obtained through visual-based navigation (**right plots**). Two different kind of trajectories and distributions of landmarks are simulated: (**upper plots**) a circular trajectory, (**lower plots**) a figure-eight-like trajectory. The GPS signal was used only during the initialization period. The actual trajectory is shown in black. The estimates are shown in blue.

Figure 9 illustrates two cases of simulation: (a) The quadrotor was commanded to take off from the ground and then to follow a circular trajectory with constant altitude. The environment is composed by 3D points, uniformly distributed over the ground, which emulate visual landmarks; (b) The quadrotor was commanded to take off from the ground and then to follow a figure-eight-like trajectory with constant altitude. The environment is composed by 3D points, randomly distributed over the ground.

In simulations, it is assumed that the camera can detect and track visual features, avoiding the data association problem. Furthermore, the problem of the influence of the estimates on the control system was not considered. In other words, an almost perfect control over the vehicle is assumed.

**Figure 10:**
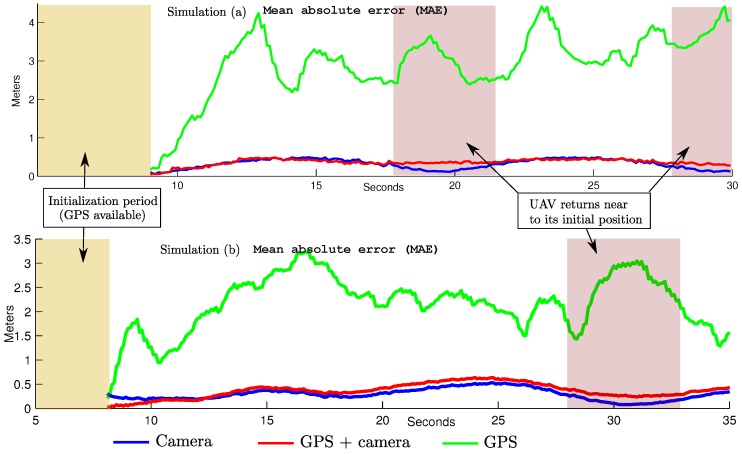
Mean absolute error (MAE) in position computed from two simulations (a and b) out of 20 Monte Carlo runs: (**upper plot**) simulation (a) results; (**lower plot**) simulation (b) results.

Figure 10 shows the average mean absolute error (MAE) in position, obtained after 20 Monte Carlo runs of simulation. The MAE was computed for three scenarios: (i) using only GPS to estimate position; (ii) using GPS together with camera along all of the trajectory in order to estimate position and map; (iii) using GPS only during the initialization period, and then performing visual-based navigation and mapping.

Figure 9 and Figure 10 clearly show the benefits of incorporating visual information into the system. It is important to note that the trajectory obtained relying only on the GPS was computed by incorporating GPS readings into the filter, and do not denote raw measurements. In Figure 10 it is interesting to note that the computed MAE values for the trajectories obtained through visual-based navigation exhibit the classical SLAM behaviour when the quadrotor returns near to its initial position. In this case, the error is minimized close to zero. On the other hand, when the GPS is used all the time, the MAE remains more constant. In this case, it is seen that even when the vehicle is close to its trusted position, there is some influence of the GPS errors that affect the estimation. This behaviour suggests that for trajectories performed near to a local frame of reference, and even when the GPS signal is available, it is better to navigate having more confidence in visual information than in GPS data. On the other hand, in the case of trajectories moving far away from its initial frame of reference, the use of absolute referenced data obtained from the GPS imposes an upper bound on the ever growing error, contrary to what is expected with a pure vision-based SLAM approach.

In these experiments, it is important to note that the most relevant source of error comes from the slow-time varying bias part of the GPS. In this case, some of the effects of this bias can be tackled by the model in Equation (5) by means of increasing the measurement noise covariance matrix. On the other hand, it was found that increasing this measurement matrix too much can affect the convergence of initial features depth. A future work could be, for instance, to develop an adaptive criteria to fuse GPS data, or also to extend the method in order to explicitly estimate the slow-varying bias of the GPS.

### 4.2. Experiments with Real Data

**Figure 11:**
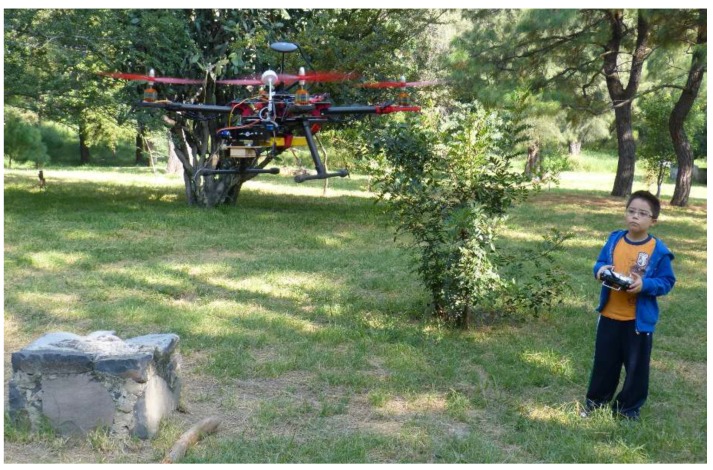
A park was used as flight field. Data obtained from the sensors of a radio-controlled quadrotor has been used to test the proposed method. The eight year-old first author’s son was in charge of piloting the flying vehicle.

A custom-built quadrotor is used to perform experiments with real data. The vehicle is equipped with an Ardupilot unit as flight controller [37], a NEO-M8N GPS unit, a radio telemetry unit 3 DR 915 Mhz, a DX201 DPS camera with wide angle lens, and a 5.8 GHz video transmitter. In experiments, the quadrotor has been manually radio-controlled (see Figure 11).

A custom-built C++ application running on a laptop has been used to capture data from the vehicle, which were received via MAVLINK protocol [38], as well as capturing the digitalized video signal transmitted from the vehicle. The data captured from the GPS, AHRS, and frames from the camera were synchronized and stored in a dataset. The frames with a resolution of 320 × 240 pixels, in gray scale, were captured at 26 fps. The flights of the quadrotor were conducted in a open area of a park surrounded by trees, see Figure 11. The surface of the field is mainly flat and composed by grass and dirt, but the experimental environment also included some small structures and plants. An average of 8–9 GPS satellites were visible at the same time.

In experiments, in order to have an external reference of the flight trajectory to evaluate the performance of the proposed method, four marks were placed in the floor, forming a square of known dimensions (see Figure 4). Then, a perspective on 4-point (P4P) technique [39] was applied to each frame in order to compute the relative position of the camera with respect to this known reference. It is important to note that the trajectory obtained by the above technique should not be considered as a perfect reference of ground-truth. However, this approach was very helpful to have a fully independent reference of flight for evaluation purposes. Finally, the MATLAB implementation of the proposed method has been executed offline for all the dataset in order to estimate the flight trajectory and the map of the environment.

**Figure 12:**
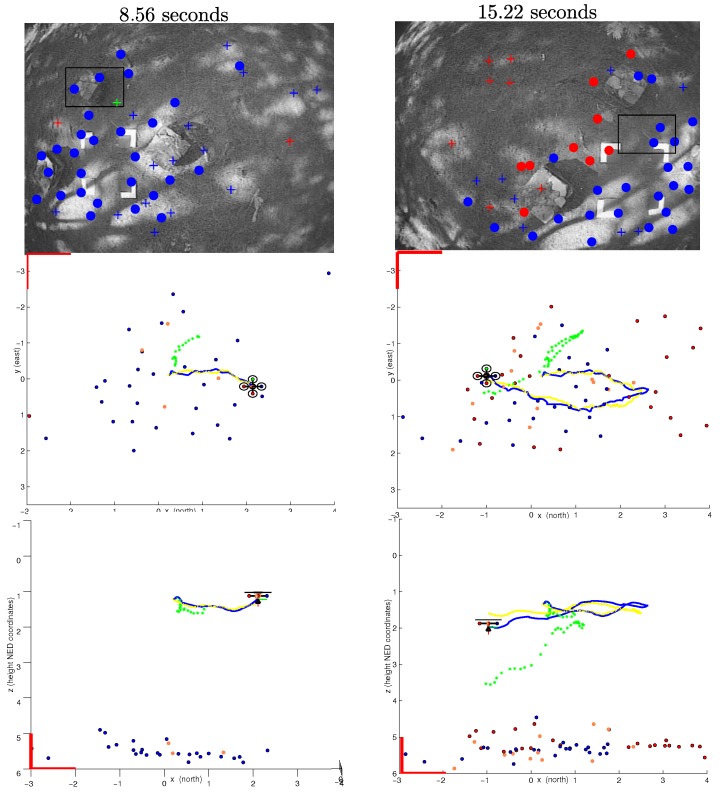
Estimated trajectory and map corresponding to two different instants of time during periods of visual-based navigation: (**upper plots**) real images at 8.56 s and 15.22 s of flight; (**middle plots**) zenital view of maps and estimated trajectories at 8.56 s and 15.22 s of flight; (**lower plots**) sectional view of maps and estimated trajectories at 8.56 s and 15.22 s of flight. The estimated trajectory is indicated in blue. The P4P visual reference is indicated in yellow. GPS position measurements are indicated in green. Comparing visual features with the estimated map, it can be appreciated that the physical structure of the environment is partially recovered.

An initial period of flight was considered for initialization purposes, as explained in Section 3.4. Figure 12 shows two different instances of a flight trajectory. For this test, the GPS readings were fused into the system only at the initialization period; after that, the position of the vehicle and the map of the environment were recovered using visual information. Since the beginning of the flight (left plots), it can be clearly appreciated how the GPS readings diverge from the actual trajectory. Several features have been included into the map just after a few seconds of flight (right plots).

**Figure 13:**
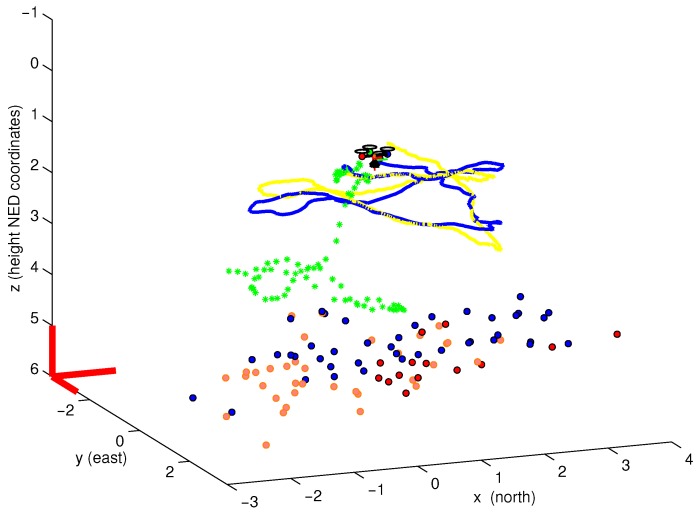
3D plot of the estimated map and trajectory obtained in visual-based navigation mode. Considering the trajectory obtained by the P4P visual technique as a reference, it can be clearly appreciated that GPS is unreliable to estimate position when fine manoeuvres are performed.

Figure 13 shows a 3D perspective of the estimated map and trajectory after 30 s of flight. In this test, a good concordance between the estimated trajectory and the P4P visual reference were obtained, especially if it is compared with the GPS trajectory.

In order to gain more insight about the performance of the proposed method, the same three experimental variants used in simulations were computed, but in this case with real data: (i) GPS; (ii) GPS + camera; (iii) camera (GPS only at the initialization). In this comparison, all the results were obtained averaging ten executions of each method. Is important to note that those averages are computed because the method is not deterministic since the search and detection of new candidate points is conducted in a random manner over the images (Section 3.3.1). The P4P visual reference was used as ground-truth. The number of visual features being tracked at each frame can affect the performance of monocular SLAM methods. For this reason, the methods were tested by setting two different values of minimum distance (M.D.) between the visual features being tracked. In this case, the bigger the value, the lesser the number of visual features that can be tracked.

**Figure 14:**
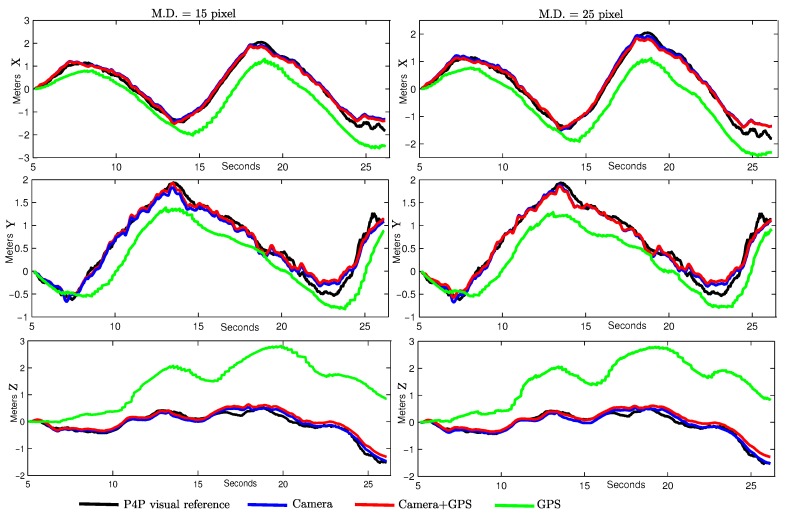
Estimated average of position expressed in coordinates for a minimum distance of 15 pixels: north (**left upper plot**), east (**left middle plot**), and down (**left lower plot**), and for a minimum distance of 25 pixels: north (**right upper plot**), east (**right middle plot**), and down (**right lower plot**). A period of 5 s of initialization was considered where the GPS was available.

Figure 14 shows the progression over time for each case. A separate plot for each coordinate (north, east, and down) is presented. Table 1 gives a numerical summary of the results obtained in this experimental comparison with real data. These results confirm the results obtained through simulations. For trajectories estimated using only GPS data, the high average MAE in position makes this approach not suitable for its use as feedback to control fine manoeuvres. In this particular case, it is easy to see that the major source of error comes from the altitude computed by the GPS (see Figure 14, lower plots). Additional sensors (e.g., a barometer) can be used to mitigate this particular error. However, the error in the horizontal plane (north–east) can be still critical for certain applications. In this sense, the benefits obtained by including visual information into the system are evident.

As it could be expected, the number of map features increases considerably as the minimum distance between visual points is decremented. However, it is interesting to remark that, at least for these experiments, there was no important improvement in error reduction. Regarding the use of the GPS altogether with monocular vision, a slightly better concordance was obtained between the P4P reference and trajectory estimated avoiding the GPS data (after the initialization). These results still suggest that, at least for small environments, it could be better to rely more on visual information than on GPS data after the initialization period.

The feasibility to implement monocular SLAM methods in real-time has been widely studied in the past. In particular, since the work of Davison in 2003 [32], the feasibility for EKF-based methods was shown for maps composed of up to 100 features using standard hardware. Later, in [29], it was shown that filter-based methods might be beneficial if limited processing power is available. Even real-time performance has been demonstrated for relatively high computation demanding techniques as the optimization-based method proposed in [40]. In the application proposed in this work, it can be seen (Table 1) that the number of features that are maintained into the system state (even for the low M.D.) are considerably below an upper bound that should allow a real-time performance, for instance by implementing the algorithm in C or C++ languages.

## 5. Conclusions

In this work, a vision-based navigation and mapping system with application to unmanned aerial vehicles has been presented. The visual information is obtained with a camera integrated in the flying vehicle pointing to the ground. The proposed scheme is closely related to monocular SLAM systems where a unique camera is used to concurrently estimate a map of visual features as well as the trajectory of the camera. As a difference from the purely monocular SLAM approaches, in this work a multi-sensor scheme is followed in order to take advantage of the set of sensors commonly available in UAVs in order to overcome some technical difficulties associated with monocular SLAM systems.

When a monocular camera is used, depth information cannot be retrieved in a single frame. In this work, a novel method is developed with this purpose. The proposed approach is based on a stochastic technique of triangulation to estimate features depth. Another important challenge that arises with the use of monocular vision comes with the fact that the metric scale of the environment can be only retrieved with a known factor if no additional information is incorporated into the system. In this work, the GPS readings are used during an initial short period of time in order to set the metric scale of estimation. After this period, the system operates relying uniquely on visual information to estimate the location of the vehicle.

Due to the highly noisy nature of the GPS measurements, it is unreliable to work only with filtered GPS data in order to obtain an accurate estimation of position to perform fine manoeuvres. In this case, visual information is incorporated into the system in order to refine such estimations.

The experimental results obtained through simulations as well as with real data suggest the following and relevant conclusions: (i) the integration into the system of very noisy GPS measurements during an initial short period is enough to recover the metric scale of the world; (ii) for flight trajectories performed near to the origin of the navigation frame of reference it is better to avoid integration of GPS measurements after the initialization period.

## Figures and Tables

**Table 1:** Numerical results in real data experiments; (i) M.D. stands for minimum distance between features (in pixels units); (ii) N.O.F. stands for average number of features maintained into the system state; (iii) aMAE stands for average mean absolute error (in meters).

	M.D. (15p)	M.D. (15p)	M.D. (25p)	M.D. (25p)
**Method**	**N.O.F.**	**aMAE (m)**	**N.O.F. (s)**	**aMAE (m)**
GPS	-	1.70 ± 0.77*σ*	-	1.70 ± 0.77*σ*
Camera + GPS	56.4 ± 10.2*σ*	0.21 ± 0.11*σ*	30.9 ± 4.9*σ*	0.22 ± 0.10*σ*
Camera	57.9 ± 9.3*σ*	0.20 ± 0.09*σ*	30.9 ± 5.6*σ*	0.20 ± 0.08*σ*

## Nomenclature

$p^{N}$	3D point, defined in euclidean coordinates, expressed in frame ${}^{N}$
$p^{C}$	3D point, defined in euclidean coordinates, expressed in frame ${}^{C}$
u,v	Undistorted pixel coordinates of a visual feature
$u_{d},v_{d}$	Distorted pixel coordinates of a visual feature
$f,u_{0},v_{0},k_{1},..k_{n}$	Intrinsic parameters of the camera
$R^{NC}$	Rotation matrix from navigation to camera frame
$t_{c}^{N}$	Position of the camera optical center expressed in the navigation frame
$h^{C}$	Vector pointing from $t_{c}^{N}$ to $P^{C}$ expressed in frame ${}^{C}$
$y_{a}^{N}$	Attitude measured
$a^{N}$	Actual attitude
$\phi_{v},\theta_{v},\psi_{v}$	Roll, pitch, and yaw of the vehicle
$v_{a}$	Modelled Gaussian white noise in attitude
$\sigma_{a}^{2}$	Attitude measurement variance
$y_{r}^{N}$	GPS position measurement
$v_{r}$	Modelled Gaussian white noise in position
$\sigma_{r}^{2}$	Position measurement variance
*x*	Augmented system state
*P*	System state covariance matrix
$x_{v}$	State of the vehicle
$q^{NR}$	Quaternion representing the orientation of the vehicle
$\omega^{R}$	Angular velocity of the vehicle
$r^{N}$	Vehicle position
$v^{N}$	Lineal velocity of the vehicle
$y_{i}^{N}$	Map feature
$x_{i},y_{i},z_{i}$	Euclidean coordinates of features
$V^{N}$	Linear velocity impulse
$\Omega^{C}$	Angular velocity impulse
$\sigma_{v}^{2}\;$	Linear velocity impulse variance
$\sigma_{\omega }^{2}\;$	Angular velocity impulse variance
*Q*	Process noise covariance matrix
$\nabla F_{x}$	Jacobian of the prediction model with respect to the system state
$\nabla F_{u}$	Jacobian of the prediction model with respect to the unknown inputs
$c_{l}$	Data stored for each candidate point
$y_{c_{i}}$	3D semi-line defined by a candidate point
$t_{c_{0}}^{N}$	Camera position when the candidate point was first observed
$\theta_{0},\phi_{0}$	Azimuth and elevation of the candidate point when it was first observed
$z_{uv}$	Visual point location
$h^{N}$	Vector pointing from $t_{c_{0}}^{N}$ to $P^{N}$ expressed in frame ${}^{C}$
$P_{y_{c_{i}}}$	Covariance matrix of $y_{c_{i}}$
$\nabla Y_{c_{i}}$	Jacobian of the function $y_{c_{i}}$ with respect to the system state and visual measurement
*d*	Feature depth
*e*	Epipolar point
$S_{c}$	Elliptical region of search of candidate points
$\alpha_{c}$	Orientation of the ellipse $S_{c}$
a,b	Major and minor axis of the ellipse $S_{c}$
*α*	Parallax of the candidate point
$e_{l}$	Displacement of the camera from its first observation to its current position
$y_{new}$	New feature to be added to the system state
$P_{y_{new}}$	Covariance matrix which models the uncertainty of $y_{new}$
∇Y	Jacobian of the function $y_{new}$ with respect to $c_{l}$ and *d*
$\sigma_{d}^{2}$	Modelled uncertainty associated with the process of depth estimation
*S*	Innovation covariance matrix
*K*	Kalman gain
*ξ*	Measurement noise covariance matrix
$\sigma_{uv}^{2}$	Visual measurement variance
*z*	Measurement vector
*h*	Predicted measurement vector
$h_{i}$	Measurement prediction model for the *i* feature
∇H	Jacobian of the function *h* with respect to the system state *x*
$h_{a}$	Measurement prediction model of attitude
$h_{r}$	Measurement prediction model of position
$P_{y_{i}}$	sub-matrix of *P* corresponding to a feature $y_{i}$
